# Loving memories of Dr. Ko Shimamoto

**DOI:** 10.1186/1939-8433-6-34

**Published:** 2013-12-10

**Authors:** Guo-Liang Wang, Jan Leach, Pamela Ronald, Hei Leung

**Affiliations:** Department of Plant Pathology, Ohio State University, Columbus, OH 43210 USA; Department of Bioagricultural Sciences and Pest Management, Colorado State University, Fort Collins, CO 80523 USA; Department of Plant Pathology, University of California at Davis, Davis, CA 95616 USA; International Rice Research Institute, Los Banos, Philippines

The plant community was deeply saddened by the sudden death of Dr. Ko Shimamoto on September 28, 2013. Dr. Shimamoto was a professor of plant molecular genetics at the Nara Institute of Science and Technology (NAIST), Nara, Japan. He is survived by his wife Taiko Shimamoto and two adult sons.

**Figure 1:**
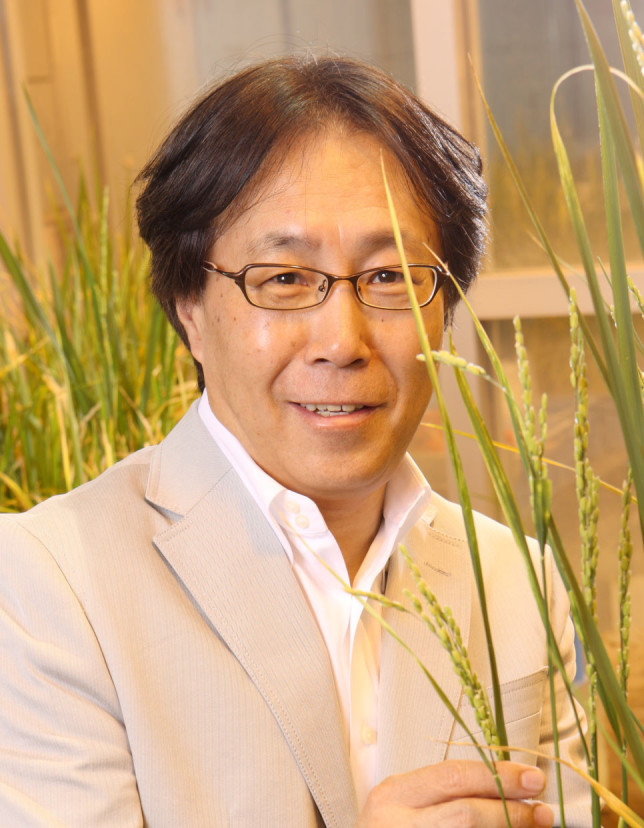
Figure 1

Dr. Shimamoto was born on October 19, 1949 in Wakayama Prefecture, Japan. He received his B.S. degree in 1974 from Kyoto University and his Ph.D. degree in Genetics from the University of Wisconsin-Madison in 1980. From 1980-83, he carried out postdoctoral training at the Friedrich Miescher Institute in Basel, Switzerland. In 1983, he joined the Plantech Research Institute established by Mitsubishi Chemical Corporation and was promoted to a senior scientist in 1988. In 1994 he was offered a professor position in the Graduate School of Biological Sciences, NAIST where he was head of the Laboratory of Plant Molecular Genetics.

Dr. Shimamoto developed a strong interest in plant genetics when he was an undergraduate student at Kyoto University. He was fascinated by the novel and powerful molecular genetic techniques. He applied these methods to the study of somatic cell genetics of maize when he was a PhD student in Madison. During his postdoctoral training in Basel, he continued working on maize cell genetics and auxotrophic mutants of plants. After returning to Japan in 1983, Ko began his work on rice and developed rice transformation methods in 1989. During his 20-year career at NAIST, he worked in various areas of rice molecular biology including disease resistance, flower development, functional genomics, and pre-mRNA splicing.

Dr. Shimamoto made many seminal and breakthrough achievements in plant molecular biology, particularly in rice transformation, flowering and disease resistance. He was the first to successfully introduce transgenes into rice in 1989, paving the way for the genetic improvement of this important staple food for half of the world’s population. Most of the today’s rice geneticists were inspired by his work and benefited from his personal and enthusiastic encouragement. “Ko was always ahead of his times, one of the first to propose rice as an experimental system that caught my attention and of many others” said Andy Pereira.

In 2007, he discovered the flowering hormone “florigen”, which had been a mystery for over 70 years. Furthermore, in 2011, he identified the florigen receptor, revealing the control mechanism for flowering in rice. Dr. Shimamoto also made significant contributions to our understanding of innate immunity of rice to pathogens. In 1999, he identified OsRAC1, an important molecule controlling disease resistance in rice, He subsequently discovered several OsRAC1-assocatied proteins that play roles in PAMP- and effector-triggered immunities. Based on these results, he proposed the defensome complex model to illustrate the immune signaling networks in rice. Dr. Shimamoto developed useful tools for plant functional genomics, such as the pANDA vectors developed in his laboratory that are being used worldwide by many plant scientists. His many seminal papers are published in high-impact international journals like Nature, Science, PNAS and the Plant Cell.

Dr. Shimamoto devoted himself to training, teaching and educational activities. Many of his graduate students play leading roles in different fields of plant sciences in Japan and abroad. During 2007–2011, he lead the Global Center of Excellence (COE) Program at NAIST’s Graduate School of Biological Sciences, a program that enabled young scientists in Japan, China and the USA to visit each other and exchange new research findings. This project led to close collaborations with leading universities and research institutes in the three countries.

Dr. Shimamoto was an amazing citizen of the plant science community. He served as an Editor for "Plant Cell Reports" (1992-1995), "The Plant Journal" (1995-1998), "Plant and Cell Physiology" (2000-2013) and "Plant Physiology" (2000-2013) and as a member of the Advisory Editorial Board of "Trends in Plant Sciences". He was the organizer of the International Congress of Molecular Plant-Microbe Interactions held in Kyoto, Japan in 2012.

**Figure 2:**
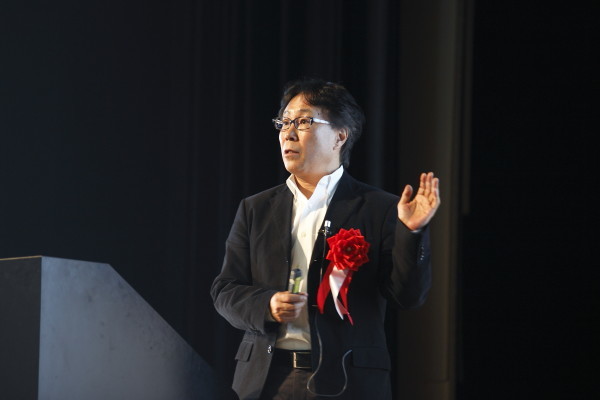
Figure 2

Dr. Shimamoto received the Distinguished Research Award from the Genetics Society of Japan (1990), the Society Award from Japanese Society of Breeding (1993), and Kihara Memorial Foundation Prize (2000), the Prize for Science and Technology from Japan’s Ministry of Education, Culture, Sports, Science, and Technology in 2011. Last year, he was awarded the prestigious Purple Ribbon Medal of Honor from the Japanese Government.

Dr. Shimamoto was a superb violin player. When he was a child, his parents asked him to practice violin 30 min every day after school before going out to play with his friends. A lab tradition was for Dr. Shimamoto to play violin at parties held after the fall rice harvest. Many of us remember Dr. Shimamoto happily dancing to celebrate the end of the productive International Symposia on Molecular Plant-Microbe Interactions in 2012. He liked to ski and read books (his favorite writer was Haruki Murakami) when he had time to relax.

Dr. Shimamoto’s death is a tremendous loss to the plant community. He will be always remembered as an outstanding scientist, patient mentor, excellent collaborator, and a loving husband and father. In an email statement, Takeshi Itoh reflects the loss that all of us feel, “I still can't believe we can't see him again”. (Photo credit: Nara Institute of Science and Technology).

## Authors’ original submitted files for images

Below are the links to the authors’ original submitted files for images. Authors’ original file for figure 1 Authors’ original file for figure 2

