# Supplementary figures and images for: Loving memories of Dr. Ko Shimamoto
Source: Rice (N Y). 2013 Dec 10;6:34. doi: 10.1186/1939-8433-6-34 (PMC4883726; doi:10.1186/1939-8433-6-34)

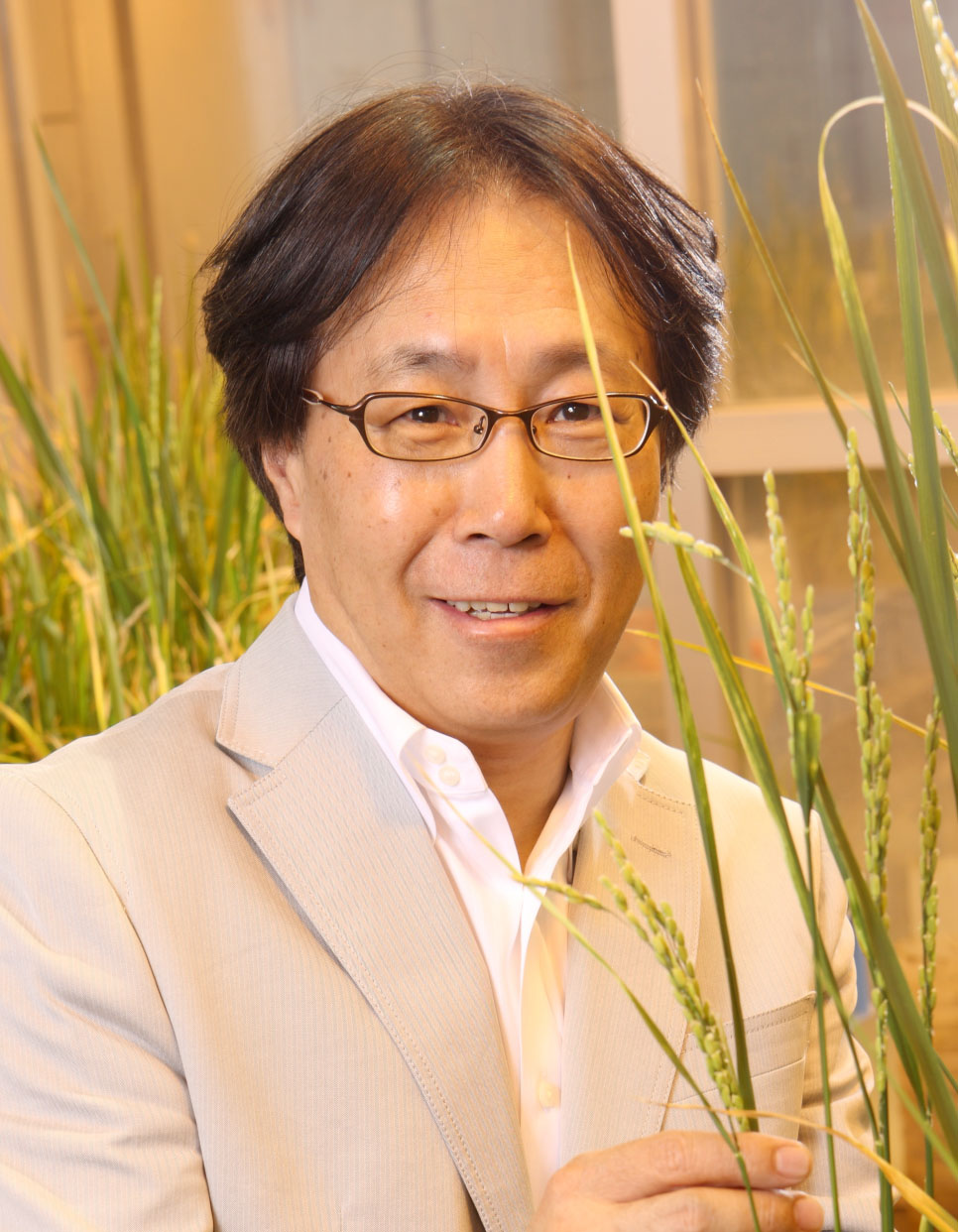

Supplement: Supplementary file 1 — Authors’ original file for figure 1 [file 12284_2013_68_MOESM1_ESM.jpeg]

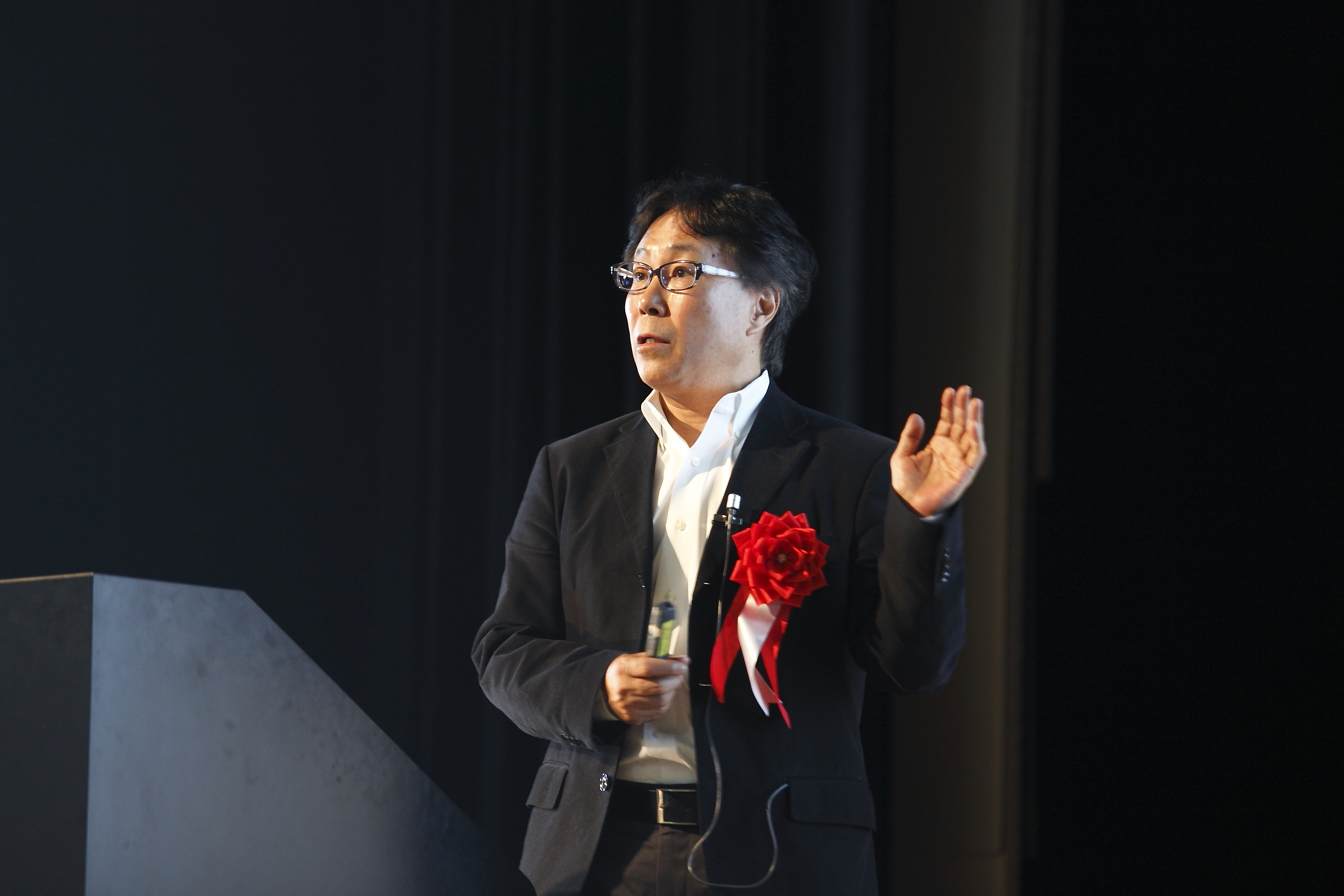

Supplement: Supplementary file 2 — Authors’ original file for figure 2 [file 12284_2013_68_MOESM2_ESM.jpeg]
